# Predicting infection with COVID-19 disease using logistic regression model in Karak City, Jordan

**DOI:** 10.12688/f1000research.129799.1

**Published:** 2023-02-02

**Authors:** Anas Khaleel, Wael Abu Dayyih, Lina AlTamimi, Liana Dalaeen, Zainab Zakaraya, Alhareth Ahmad, Baker Albadareen, Abdallah Ahmed Elbakkoush

**Affiliations:** 1 Department of Pharmacology and Biomedical Sciences, Faculty of Pharmacy, Petra University, Amman, Jordan; 2 Faculty of Pharmacy, Mutah University, Karak-61710, Jordan; 3 Department of Clinical Pharmacy, Faculty of Pharmacy, Zarqa University, Zarqa, Jordan; 4 Biopharmaceutics and Clinical Pharmacy Department, Faculty of Pharmacy, AL-Ahliyya Amman University, Amman, Jordan; 5 Department of Applied Mathematics, Faculty of Applied Science, Palestine Technical University – Kadoorie, Tulkarm, Palestinian Territory; 6 Institute of Biomedical Informatics, College of Medical Science and Technology, Taipei Medical University, Taipei, Taiwan; 7 Li Chen Biomedical Company, Taipei, Taiwan

**Keywords:** COVID-19, Google Sheet, Logistic regression model, Sex, Age, Smoking

## Abstract

**Background**: On March 2020, World Health Organization (WHO) labeled coronavirus disease 2019 (COVID-19) as a pandemic. COVID-19 has rapidly increased in Jordan which resulted in the announcement of the emergency state on March 19th, 2020. Despite the variety of research being reported, there is no agreement on the variables that predict COVID-19 infection. We have analyzed the data collected from Karak city citizens to predict the probability of infection with COVID-19 using binary logistic regression model.

**Methods:** Based on data collected by Google sheet of COVID-19 infected and non-infected persons in Karak city, analysis was applied to predict COVID-19 infection probability using a binary logistic regression model.

**Results:** The ultimate logistic regression model provides the formula of COVID-19 infection probability based on sex and age variables.

**Conclusions:** Given a person's age and sex, the final model presented in this study can be used to calculate the probability of infection with COVID-19 in Karak city. This could help aid health-care management and policymakers in properly planning and allocating health-care resources.

## Introduction

In December 2019, coronavirus disease 2019 (COVID-19) was first reported in Wuhan city, China. ^1^^,^^2^ It wasn't long before it was determined that severe acute respiratory syndrome coronavirus 2 (SARS-CoV-2) causes COVID-19. ^3^ This virus spread quickly all over the world and was declared by the World Health Organization as a pandemic. ^4^

Most of the studies have concentrated on the outbreak in China since the first reported cases were published, including the disease's transmission, risk factors for infection, and biological features of the virus using different statistical models as can be seen in literatures. ^5^^–^^9^ An exponential model was used to predict the number of infected people in Italy based on the data reported by the Italian Health Ministry. ^10^ Maleki *et al* examined the data of confirmed and recovered COVID-19 cases using a set of two-piece scale mixture normal distributions models. ^11^

The Susceptible-Infective-Recovered (SIR) model was used to anticipate the characteristics of COVID-19 cases in China. ^12^ Caccavo *et al* proposed a modified Susceptible, Infected, Removed, and Dead (SIRD) model to estimate how the COVID-19 outbreaks in China and Italy will develop. ^13^ In another work a deep-learning algorithm called long short-term memory (LSTM) was used to anticipate COVID-19 cases in Iran. ^14^

The first diagnosed COVID-19 case in Jordan was reported in March 2020, a Jordanian citizen who had returned from Italy. ^15^ At the end of 2020 Jordan reported more than 271,000 COVID-19 verified cases and more than 3,500 fatalities. ^16^

In this work we used a binary regression model to fit the data collected from Karak city citizens by Google sheet. We were able to build a final equation that can predict the probability of infection with COVID-19 in Karak city based on sex and age variables.

## Methods

### Ethical considerations

This study was approved by ethical committee in Mutah University and University of Petra (MUTAH-UOP no.:20219091) on 9 ^th^ September 2021. Informed consent was obtained from the study subjects, via a question at the start of the survey: “I agree to answer questions: yes/no”. Those who refused to answer or did not want to continue answering the questions were allowed to opt out any time. The ethical approval number was posted on the top of questionnaire first page and an email and telephone number of the principal investigator was also posted in case of any question or inquiry.

### Study design

A structured questionnaire via google sheet was used to gather information from Karak city residents. The information was collected from September 10 ^th^ to the end of October 2021. The survey has employed a variety of demographic variables including sex, age, job, smoking, chronic disease, yearly flu injection, and infected with COVID-19 before vaccination. All participants received an explanation of the questionnaire's goals and objectives at the outset of the survey.

The questionnaire was written by the authors in Arabic language, translated two ways and provided as *Extended data.* ^26^ The questionnaires' reliability and validity were not performed, and the authors couldn’t follow up the participants since they didn’t provide their personal contact information.

In order to avoid any potential bias in our study the survey questions were clear, direct, and the sequence order of the questions were designed in a way to avoid influencing the participants’ answers. Moreover, we didn’t limit the time period for the participants to complete the questionnaire and the selection of participants was random.

### Sample

In this work the Raosoft sample size calculator was used to determine the expected sample size. ^17^ Based on a 50% response rate, a 95% confidence interval, and a 5% margin of error, the sample size was estimated. The maximum sample size needed is 377. Consequently, this research used a practical sample of 386 persons out of 402 participants. A total of 16 participants were excluded from the study due to missing data. Being an adult (older than 18 years old) and residing in Karak city during the study were requirements for inclusion.

The research method employed was a cross-sectional study design with random convenience sampling. Using Google Forms, an anonymous survey was posted online and shared on popular social media sites including Facebook and WhatsApp.

### Data analysis

The IBM SPSS statistics 22 software was used to evaluate the data. The binary logistic regression model and the likelihood ratio (LR) chi-square test were used in the analysis. A significant P-value < 0.05 was statistically considered.

## Results

A total of 323 of the 386 participants that responded to the survey were women, while 63 of the participants were men. ^25^ In this study there were two categories for age: one related to participants aged less than or equal 45 years old (<=45) and the other one related to participants aged over 45 years old (>45). This age cut value has been chosen based on a study was done in United States in 2020. The results of this study showed that the number of infected people with COVID-19 was higher among those aged 40 – 49 years old. ^18^ In our study we have calculated the median age of that interval which results in the age cut value 45 years old.

The statistical analysis of the collected data for different geographic variables shows that 295 (76.4%) participants were aged less than or equal 45 years old, while 91 (23.6%) persons were aged over 45 years old. The number of participants who had non-medical job was found to be 166 (43%) from the whole participants, while 77 (19.9%) are working in the medical field, in addition to 69 (17.9%) students, and finally a total of 74 (19.2%) are unemployed.

Moreover, the number of participants who smoke in our sample is 68 (17.6%), the former smokers were 317 (82.1%), and 1 participant was a non-smoker. In total, 91 (24.6%) participants had a chronic disease, and a total of 291 (75.4%) persons didn’t suffer from any disease. The number of the persons who took the yearly flu vaccine were found to be 40 (10.4%) persons, and 346 (89.6%) didn’t take the yearly flu vaccine. All demographic variables of the participants in this study are shown below in Table 1.

**Table 1.** Participant’s characteristics.

Demographic variable		Number (%)
**Sex**	Female	323 (83.8%)
	Male	63 (16.4%)
**Age (years)**	<=45	295 (76.4%)
	>45	91 (23.6%)
**Job**	Medical	77 (19.9%)
	Non-medical	166 (43%)
	Student	69 (17.9%)
	Unemployed	74 (19.2%)
**Smoker**	Yes	68 (17.6%)
	No	1 (0.3%)
	Former	317 (82.1%)
**Chronic disease**	Yes	91 (24.6%)
	No	291 (75.4%)
**Yearly flu injection**	Yes	40 (10.4%)
	No	346 (89.6%)

The statistical analysis of our sample shows that 257 of the participants have been infected with COVID-19 and they are distributed with respect to demographics variables as shown in Table 2 below.

**Table 2.** Distribution of infected participants with COVID-19 versus demographics variables.

Demographic variable		Infected No. (%)	Total (%)
**Sex**	Female	223 (86.8%)	257(100%)
	Male	34 (13.2%)	
**Age (years)**	<=45	188 (73.2%)	257(100%)
	>45	69 (26.8%)	
**Job**	Medical	52 (20.2%)	257(100%)
	Non-medical	120 (46.7%)	
	Student	41 (16%)	
	Unemployed	44 (17.1%)	
**Smoker**	Yes	45 (17.5%)	257(100%)
	No	1 (0.4%)	
	Former	211 (82.1%)	
**Chronic disease**	Yes	60 (23.3%)	257(100%)
	No	197 (76.7%)	
**Yearly flu injection**	Yes	26 (10.1%)	257(100%)
	No	231 (89.9%)	

Table 2, shows that among the 275 persons infected with COVID-19, 223 (86.8%) were women, 167 of them aged <= 45 years old and 56 of them aged over 45 years old. For the 34 (13.2%) infected men 21 of them were aged <= 45 years old while 13 were aged over 45 years old. This leads to a total of 188 (73.2%) of the infected participants aged <= 45 years old, and 69 (26.8%) aged over 45 years old.

The number of infected participants working in the medical field was 52 (20.2%) (45 women and 7 men). Among the infected there were 120 (46.7%) participants with a non-medical job (101 women and 19 men), 41 (16%) were infected students (35 women and 6 men), and 44 (17.5%) were unemployed infected participants (42 women and 2 men). Furthermore, the total number of infected participants who are former smokers were 211 (82.1%) (199 women and 12 men), while 45 (17.5%) (23 women and 22 men) were infected smokers.

Overall, 60 (23.3%) (51 women and 9 men) were infected participants with a chronic disease, 197 (76.7%) (172 women and 25 men) were infected without any disease. Moreover, 26 (10.1%) (18 women and 8 men) participants were flu vaccinated and infected with COVID-19, while 231 (89.9%) (205 women and 26 men) was infected with COVID-19 and didn’t take flu vaccine.

All the demographic variables in this study were tested using LR chi-square test to determine the probability of COVID-19 infection predictors. The results show that sex and age of the participants are the significant demographic infection predictors. Table 3 provides counts and ratios between these predictor variables.

**Table 3.** Sample data distribution based on the variables sex and age.

			age level > 45		
			age<=45	age>45	Total
**Sex2_0**	**female**	**Count**	252	71	323
		% Within Sex2_0	78.0%	22.0%	100.0%
		% Within age level > 45	85.4%	78.0%	83.7%
	**male**	**Count**	43	20	63
		% Within Sex2_0	68.3%	31.7%	100.0%
		% Within age level > 45	14.6%	22.0%	16.3%
**Total**		**Count**	295	91	386
		% Within Sex2_0	76.4%	23.6%	100.0%
		% Within age level > 45	100.0%	100.0%	100.0%

Among the 295 participants aged <= 45 years old 252 (78%) were women and 43 (68.3%) of them were men. For the 91 participants who aged > 45 years old 71 (22%) of them were women and 20 (31.7%) were men.

In our binary logistical regression model, we have used all predictor variables (sex and age). The results of the model are shown below in Table 4.

**Table 4.** Logistic regression coefficients and their tests.

Variables	B	Wald	P-value	Exp (B)
Sex2_0	-0.716	6.307	0.012	0.489
Age 45	0.648	5.438	0.020	1.911
Constant	0.674	26.769	0.000	1.963

Coefficients values of the model and their statistical significance P-value were obtained by ‘Enter logistical regression method’. LR chi-square test was applied to evaluate the overall model fit and to test the significant coefficients. The B coefficient values were found to be (-0.716, 0.48), while the Wald Statistics values are (6.307, 5.438) for sex and age respectively. The exponentiated logistic coefficient (Exp (B)) shows the values of 0.489 for sex and 1.911 for age cut value 45 years old.

Our model in this work uses two indicators to measure model fineness percentage; Cox & Snell R Square (R ^2^ = 0.028) and Nagelkerke R Square (R ^2^ = 0.039). Although the R ^2^ values are too small, it indicated a weak relationship. This value explanted that out model contributes about 4% of the COVID-19 infection probability as illustrated in Table 5 below. It is important to mention that the Cox & Snell R Square indicator commonly produced underestimates the real value. ^19^^,^^20^

**Table 5.** Logistic regression model summary.

Step	-2 Log likelihood	Cox & Snell R Square	Nagelkerke R Square
1	480.878 ^a^	0.028	0.039

This research aimed to predict the probability of infection with COVID-19 in Karak city. Using the final logistic regression model data presented in Table 4 results, the formula of COVID-19 infection probability (P _infected_) is given as:

$$P_{\text{infected}}=\frac{e^{0.674-0.716*\text{Gender}+0.648*\mathrm{Age}}}{1+e^{0.674-0.716*\text{Gender}+0.648*\mathrm{Age}}}$$ (1)

The probability of infection can be calculated by substituting the values for sex and age in equation (1).

## Discussion

According to the results obtained from Table 2 the number of infected women with COVID-19 was 223 (86.8%) and the number of infected men was 34 (13.2%). This difference is in agreement with the results of a study in the United States recorded from January to May 2020, where the number of infected women with COVID-19 was 51.1%, while the number of infected males was 48.9%. ^18^

In another study, infection rates for COVID-19 were reconstructed by age and sex using data from different European countries. ^21^ The results show in all the analyzed countries, the chance of infection with COVID-19 among women increases more sharply after age 20 until late 50s. ^21^

These results support the ones obtained in our study since among the 188 (73.2%) participants aged <= 45 years infected with COVID-19, 167 (88.8%) of them were women and 21 (11.2%) were males.

Moreover, in this work among the former and current smokers infected with COVID-19, 222 (86.7%) (199 former and 23 current) are women, while 34 (13.3%) (12 former and 22 current) are men. These results are consistent with previous studies which recognized that one of the risk factors associated with COVID-19 is smoking. ^22^^–^^24^

The results of the exponentiated logistic coefficient show that the probability of women to be infected with COVID-19 is more than men for the same age. This result is in agreement with the results obtained from our model ( equation (1)) for calculating the probability of infection with COVID-19.

In equation (1) the sex variable labeled by (0) for women, (1) for men, while the age variable labeled by (0) if age less than or equal 45 years old and labeled (1) if age is more than 45 years old. Using equation (1) we can calculate the probability of a man aged 33 years old to be infected with COVID-19 by substituting numbers for age and sex. The results lead to P _infected_ = 0.4895. Since P _infected_ is less than 0.5, indicates the man is not infected. Repeating the same calculations for a woman with the same age result in P _infected_ = 0.6624, indicates the woman is infected with COVID-19.

### Limitations of the study

A notable limitation of this study was its brief lifespan. There was no more observation to validate the model. Furthermore, we are unable to extrapolate our results to the other cities in Jordan because we only evaluated people in Karak city.

## Conclusion

Given a person's age and sex, equation (1) presented in this study can be used to calculate the probability of infection with COVID-19 in Karak city. This statistical model can be used to forecast outbreak trends. This forecast could aid health-care management and policymakers in properly planning and allocating health-care resources.

## Data Availability

Fighsare: supplementary data.xlsx. https://doi.org/10.6084/m9.figshare.21829650. ^25^ Figshare: questionnaire supplementary information.docx https://doi.org/10.6084/m9.figshare.21931731. ^26^ Data are available under the terms of the Creative Commons Attribution 4.0 International license (CC-BY 4.0).
