# Predicting infection with COVID-19 disease using logistic regression model in Karak City, Jordan

**DOI:** 10.12688/f1000research.129799.3

**Published:** 2025-07-30

**Authors:** Anas Khaleel, Wael Abu Dayyih, Lina AlTamimi, Liana Dalaeen, Zainab Zakaraya, Alhareth Ahmad, Baker Albadareen, Abdallah Ahmed Elbakkoush

**Affiliations:** 1 Department of Pharmacology and Biomedical Sciences, Faculty of Pharmacy, Petra University, Amman, Jordan; 2 Faculty of Pharmacy, Mutah University, Karak-61710, Jordan; 3 Department of Clinical Pharmacy, Faculty of Pharmacy, Zarqa University, Zarqa, Jordan; 4 Biopharmaceutics and Clinical Pharmacy Department, Faculty of Pharmacy, AL-Ahliyya Amman University, Amman, Jordan; 5 Department of Applied Mathematics, Faculty of Applied Science, Palestine Technical University – Kadoorie, Tulkarm, Palestinian Territory; 6 Li Chen Biomedical Company, Taipei, Taiwan; 7 Institute of Biomedical Informatics, College of Medical Science and Technology, Taipei Medical University, Taipei, Taiwan

**Keywords:** COVID-19, Google Sheet, Logistic regression model, Sex, Age, Smoking

## Abstract

**Background:**

On March 2020, World Health Organization (WHO) labeled coronavirus disease 2019 (COVID-19) as a pandemic. COVID-19 has rapidly increased in Jordan which resulted in the announcement of the emergency state on March 19th, 2020. Despite the variety of research being reported, there is no agreement on the variables that predict COVID-19 infection. This study aimed to test the predictors that probably contributed to the infection with COVID-19 using a binary logistic regression model.

**Methods:**

Based on data collected by Google sheet of COVID-19 infected and non-infected persons in Karak city, analysis was applied to predict COVID-19 infection probability using a binary logistic regression model.

**Results:**

A total of 386 participants have completed the questionnaire including 323 women and 63 men. Among the participants 295 (76.4%) were aged less than or equal 45 years old, and 91 (23.6%) were aged over 45 years old. Among the 386 participants a total of 275 were infected with COVID-19. The Logistic regression test was used to analyze every demographic characteristic (sex, age, job, smoking, chronic disease, yearly flu injection) in this study to find predictors of the likelihood of COVID-19 infection. The findings indicate that the participants’ sex and age are the most important demographic determinants of infection. Female gender was associated with higher infection risk compared to males (OR = 2.04, 95% CI: 1.17-3.58, p = 0.012). Participants aged >45 years had increased infection risk compared to those ≤45 years (OR = 1.91, 95% CI: 1.11-3.30, p = 0.020). Cox & Snell R Square (R2 = 0.028) and Nagelkerke R Square (R2 = 0.039) indicators were used to measure model fineness with a significant P-value < 0.05.

**Conclusions:**

Given a person’s age and sex, the final model presented in this study can be used to calculate the probability of infection with COVID-19 in Karak city. This could help aid health-care management and policymakers in properly planning and allocating health-care resources.

## Introduction

Based on the extensive research by Dr. Huyut and colleagues, routine blood parameters serve as powerful diagnostic and prognostic tools for COVID-19 through multiple mechanisms. Machine learning models using routine blood values have achieved diagnostic accuracies exceeding 99% in large-scale studies, demonstrating their clinical utility as cost-effective alternatives to RT-PCR testing. Research demonstrates that routine blood parameters including mean corpuscular hemoglobin concentration (MCHC), mean corpuscular hemoglobin (MCH), and activated partial thromboplastin time (aPTT) can achieve 99.17% accuracy in COVID-19 diagnosis using LogNNet neural networks. Studies consistently show that elevated inflammatory markers (CRP, procalcitonin, ferritin), coagulation abnormalities (D-dimer, INR, PT), and hematological changes (lymphopenia, elevated neutrophil-to-lymphocyte ratio) predict disease severity and progression. The combination of ferritin, INR, and D-dimer demonstrates perfect mortality prediction accuracy in multiple validation studies. These routine biomarkers enable early risk stratification and facilitate rapid screening in resource-limited settings, supporting clinical decision-making for treatment prioritization; these biomarkers may serve as a potential predictor of diagnosis, prognosis and mortality of COVID-19. ^1^^–^^12^

In December 2019, coronavirus disease 2019 (COVID-19) was first reported in Wuhan city, China. ^13^^,^^14^ It wasn't long before it was determined that severe acute respiratory syndrome coronavirus 2 (SARS-CoV-2) causes COVID-19. ^15^ This virus spread quickly all over the world and was declared by the World Health Organization as a pandemic. ^16^

Most of the studies have concentrated on the outbreak in China since the first reported cases were published, including the disease's transmission, risk factors for infection, and biological features of the virus using different statistical models as can be seen in literatures. ^17^^–^^21^ An exponential model was used to predict the number of infected people in Italy based on the data reported by the Italian Health Ministry. ^22^ Maleki *et al* examined the data of confirmed and recovered COVID-19 cases using a set of two-piece scale mixture normal distributions models. ^23^

The Susceptible-Infective-Recovered (SIR) model was used to anticipate the characteristics of COVID-19 cases in China. ^24^ Caccavo *et al* proposed a modified Susceptible, Infected, Removed, and Dead (SIRD) model to estimate how the COVID-19 outbreaks in China and Italy will develop. ^25^ In another work a deep-learning algorithm called long short-term memory (LSTM) was used to anticipate COVID-19 cases in Iran. ^26^

The first diagnosed COVID-19 case in Jordan was reported in March 2020, a Jordanian citizen who had returned from Italy. ^27^ At the end of 2020 Jordan reported more than 271,000 COVID-19 verified cases and more than 3,500 fatalities. ^28^ The Covid-19 epidemic inspired numerous investigations in Jordan. For instance, health care professionals, particularly pharmacists, believed that they required training in psychological care in order to support people during local pandemics as well as themselves. ^29^ The majority of Jordan’s general public recognized how the covid disease was spread and that chronic illnesses like diabetes and aging were risk factors as well as being immunocompromised. ^30^ Odeh *et al* developed a prediction model of risk factors for complications among COVID-19 patients in Jordan. The results of the model showed that diabetes, shortness of breath, Body Mass Index (BMI), and abnormal neutrophils are the risk factors associated with COVID-19 complications. ^31^

In another study, climate indicators including average daily temperature, relative humidity, wind velocity, pressure, and concentrations of pollutants were used to predict COVID-19 active cases in the three cities in Jordan using Artificial Neural Networks (ANN) and multiple linear regression. ^32^ Moreover, Hussein et al proposed three approaches (short-term forecast (STF) model, long-term forecast (LTF) model, and hybrid forecast (HF) model) for predicting COVID-19 pandemic’s development in Jordan over a long-term period that took into account various social circumstances like everyday life, curfews, lockdowns, and vaccination. ^33^

In this work we were able to build a final equation that can predict the probability of infection with COVID-19 in Karak city based on sex and age variables.

## Methods

### Ethical considerations

This study was approved by ethical committee in Mutah University and University of Petra (MUTAH-UOP no.:20219091) on 9 ^th^ September 2021. Informed consent was obtained from the study subjects, via a question at the start of the survey: “I agree to answer questions: yes/no”. Those who refused to answer or did not want to continue answering the questions were allowed to opt out any time. The ethical approval number was posted on the top of questionnaire first page and an email and telephone number of the principal investigator was also posted in case of any question or inquiry.

### Study design

Using information from the literature a structured questionnaire via google sheet was used to gather information from Karak city residents. The information was collected from September 10 ^th^ to the end of October 2021. The survey has employed a variety of demographic variables including sex, age, job, smoking, chronic disease, yearly flu injection, and infected with COVID-19 before vaccination. All participants received an explanation of the questionnaire's goals and objectives at the outset of the survey. The specific chronic disease breakdown was: Diabetes mellitus: (19.7%), Hypertension: (73.1%), No chronic disease: (7.2%).

Hypertension was the predominant chronic condition affecting nearly three-quarters of participants, followed by diabetes in approximately one-fifth of the cohort. This high prevalence of metabolic comorbidities is clinically significant as both conditions are established risk factors for severe COVID-19 outcomes.

Key Demographics:

Mean age: 33.4 years (range 18-65)

Elderly (≥65 years): Only (0.5%)

**Clinical context highlights:**

Young adult population (88.3% under 50 years) - lower COVID-19 risk baseline

Minimal elderly representation - limits generalizability to high-risk groups

Gender distribution reflects typical healthcare engagement patterns

Age stratification shows concentration in younger age groups

**Clinical implications:**

Lower baseline COVID-19 severity risk

Potentially stronger vaccine immune responses

Limited applicability to elderly populations

Reflects Jordan’s demographic profile

We acknowledge that **standardized disease severity classification using WHO or AIIMS guidelines was not implemented** in the original study design.

**Current data limitations:**

• Our dataset contains COVID-19 infection status and symptom presence (0-6 categorical scale) but **lacks standardized severity grading**
• No WHO Clinical Progression Scale (0-10 severity scoring) was applied
• No AIIMS severity classification (Mild/Moderate/Severe/Critical) was documented
• Missing data on hospitalization requirements, oxygen therapy needs, or clinical progression tracking

**Impact on study:** This represents a **significant methodological limitation** that restricts our ability to:

• Correlate vaccine effectiveness with disease severity outcomes
• Compare findings with international studies using WHO/AIIMS criteria
• Assess clinical progression and healthcare utilization patterns

**Limitation statement for methods:** “This study did not employ standardized disease severity classification systems such as the WHO Clinical Progression Scale or AIIMS severity grading criteria. COVID-19 symptoms were documented using a categorical scale but were not systematically graded for clinical severity, limiting our ability to correlate vaccine effectiveness with standardized disease severity outcomes.”

**Future research recommendation:** Subsequent studies should implement WHO Clinical Progression Scale (0-10) or AIIMS severity grading to enable robust assessment of vaccine effectiveness across different disease severity categories and improve clinical applicability of findings.

This limitation should be clearly acknowledged in the study’s limitations section and addressed in future research designs.

### Tool development

The questionnaire was written by the authors in Arabic language, translated two ways and provided as *Extended data.* ^50^ The questionnaires' reliability and validity were not performed, and the authors couldn’t follow up the participants since they didn’t provide their personal contact information.

The demographics part asked nine questions about gender, age, marital status, geographic area, educational attainment, insurance status, smoking habits, income, and the presence of chronic condition (medications). The COVID-19 component of the survey comprised smoking, chronic disease, yearly flu injection, and infected with COVID-19 before vaccination.

In order to avoid any potential bias in our study the survey questions were clear, direct, and the sequence order of the questions were designed in a way to avoid influencing the participants’ answers. Moreover, we didn’t limit the time period for the participants to complete the questionnaire and the selection of participants was random.

### Sample

In this work the Raosoft sample size calculator was used to determine the expected sample size. ^34^ Based on a 50% response rate, a 95% confidence interval, and a 5% margin of error, the sample size was estimated. The maximum sample size needed is 377. Consequently, this research used a practical sample of 386 persons out of 402 participants. A total of 16 participants were excluded from the study due to missing data. Being an adult (older than 18 years old) and residing in Karak city during the study were requirements for inclusion.

The research method employed was a cross-sectional study design with random convenience sampling. Using Google Forms, an anonymous survey was posted online and shared on popular social media sites including Facebook and WhatsApp.

### Data analysis

The IBM SPSS statistics 22 software was used to evaluate the data. The binary logistic regression model and the Logistic regression test were used in the analysis. A significant P-value < 0.05 was statistically considered. All of the Essential Assumptions for Implementing Logistic Regression model were evaluated and determined to be valid. Variables were summarized using descriptive statistics with means ±standard deviations for continuous variables and frequencies with percentages for categorical variables. Logistic regression analysis was selected over simple chi-square testing because it enables multivariable analysis while controlling for confounding variables and provides odds ratios for risk quantification.

The purposeful selection approach was employed for variable selection, beginning with univariate analysis (p<0.25 threshold) followed by multivariable modeling. This approach was chosen over automated stepwise methods to ensure clinical relevance and model stability. Model assumptions were assessed through linearity testing and goodness-of-fit evaluation using the Hosmer-Lemeshow test.

Chi-square vs. Logistic Regression clarification:

Chi-square: Tests simple associations between categorical variables in bivariable analysis

Logistic regression: Enables multivariable analysis with simultaneous adjustment for multiple predictors and provides odds ratios for clinical interpretation

Rationale: “Logistic regression was selected to control for potential confounding variables and provide clinically interpretable odds ratios for risk assessment”

## Results

A total of 323 of the 386 participants that responded to the survey were women, while 63 of the participants were men. ^49^ In this study there were two categories for age: one related to participants aged less than or equal 45 years old (<=45) and the other one related to participants aged over 45 years old (>45). This age cut value has been chosen based on a study was done in United States in 2020. The results of this study showed that the number of infected people with COVID-19 was higher among those aged 40 – 49 years old. ^35^ In our study we have calculated the median age of that interval which results in the age cut value 45 years old.

The statistical analysis of the collected data for different geographic variables shows that 295 (76.4%) participants were aged less than or equal 45 years old, while 91 (23.6%) persons were aged over 45 years old. The number of participants who had non-medical job was found to be 166 (43%) from the whole participants, while 77 (19.9%) are working in the medical field, in addition to 69 (17.9%) students, and finally a total of 74 (19.2%) are unemployed.

Moreover, the number of participants who smoke in our sample is 68 (17.6%), the former smokers were 317 (82.1%), and 1 participant was a non-smoker. In total, 91 (24.6%) participants had a chronic disease, and a total of 291 (75.4%) persons didn’t suffer from any disease. The number of the persons who took the yearly flu vaccine were found to be 40 (10.4%) persons, and 346 (89.6%) didn’t take the yearly flu vaccine. All demographic variables of the participants in this study are shown below in Table 1.

**Table 1.** Participant’s characteristics.

Demographic variable		Number (%)
**Sex**	Female	323 (83.8%)
	Male	63 (16.4%)
**Age (years)**	<=45	295 (76.4%)
	>45	91 (23.6%)
**Job**	Medical	77 (19.9%)
	Non-medical	166 (43%)
	Student	69 (17.9%)
	Unemployed	74 (19.2%)
**Smoker**	Yes	68 (17.6%)
	No	1 (0.3%)
	Former	317 (82.1%)
**Chronic disease**	Yes	91 (24.6%)
	No	291 (75.4%)
**Yearly flu injection**	Yes	40 (10.4%)
	No	346 (89.6%)

The statistical analysis of our sample shows that 257 of the participants have been infected with COVID-19 and they are distributed with respect to demographics variables as shown in Table 2 below.

**Table 2.** Distribution of infected participants with COVID-19 versus demographics variables.

Demographic variable		Infected No. (%)	Total (%)
**Sex**	Female	223 (86.8%)	257(100%)
	Male	34 (13.2%)	
**Age (years)**	<=45	188 (73.2%)	257(100%)
	>45	69 (26.8%)	
**Job**	Medical	52 (20.2%)	257(100%)
	Non-medical	120 (46.7%)	
	Student	41 (16%)	
	Unemployed	44 (17.1%)	
**Smoker**	Yes	45 (17.5%)	257(100%)
	No	1 (0.4%)	
	Former	211 (82.1%)	
**Chronic disease**	Yes	60 (23.3%)	257(100%)
	No	197 (76.7%)	
**Yearly flu injection**	Yes	26 (10.1%)	257(100%)
	No	231 (89.9%)	

Table 2, shows that among the 275 persons infected with COVID-19, 223 (86.8%) were women, 167 of them aged <= 45 years old and 56 of them aged over 45 years old. For the 34 (13.2%) infected men 21 of them were aged <= 45 years old while 13 were aged over 45 years old. This leads to a total of 188 (73.2%) of the infected participants aged <= 45 years old, and 69 (26.8%) aged over 45 years old.

The number of infected participants working in the medical field was 52 (20.2%) (45 women and 7 men). Among the infected there were 120 (46.7%) participants with a non-medical job (101 women and 19 men), 41 (16%) were infected students (35 women and 6 men), and 44 (17.5%) were unemployed infected participants (42 women and 2 men). Furthermore, the total number of infected participants who are former smokers were 211 (82.1%) (199 women and 12 men), while 45 (17.5%) (23 women and 22 men) were infected smokers.

Overall, 60 (23.3%) (51 women and 9 men) were infected participants with a chronic disease, 197 (76.7%) (172 women and 25 men) were infected without any disease. Moreover, 26 (10.1%) (18 women and 8 men) participants were flu vaccinated and infected with COVID-19, while 231 (89.9%) (205 women and 26 men) was infected with COVID-19 and didn’t take flu vaccine.

All the demographic variables in this study were tested using Logistic regression test to determine the probability of COVID-19 infection predictors. The results show that sex and age of the participants are the significant demographic infection predictors. Table 3 provides counts and ratios between these predictor variables.

**Table 3.** Sample data distribution based on the variables sex and age.

			age level > 45		
			age<=45	age>45	Total
**Sex2_0**	**female**	**Count**	252	71	323
		% Within Sex2_0	78.0%	22.0%	100.0%
		% Within age level > 45	85.4%	78.0%	83.7%
	**male**	**Count**	43	20	63
		% Within Sex2_0	68.3%	31.7%	100.0%
		% Within age level > 45	14.6%	22.0%	16.3%
**Total**		**Count**	295	91	386
		% Within Sex2_0	76.4%	23.6%	100.0%
		% Within age level > 45	100.0%	100.0%	100.0%

Among the 295 participants aged <= 45 years old 252 (78%) were women and 43 (68.3%) of them were men. For the 91 participants who aged > 45 years old 71 (22%) of them were women and 20 (31.7%) were men.

In our binary logistical regression model, we have used all predictor variables (sex and age). The results of the model are shown below.

“In this logistic regression analysis predicting COVID-19 positive test results:

1. Sex Variable (OR = 0.489, p = 0.012):

“Patients with [specific sex category] are 51% less likely to test positive for COVID-19 compared to the reference sex category.”

2. Age Variable (OR = 1.911, p = 0.020):

“Patients aged 45 years and older are 1.91 times more likely to test positive for COVID-19 compared to patients younger than 45 years."

**Interpretation of Odds-Ratio (Exp) Coefficients**

1. Sex2_0: OR = 0.489 (p = 0.012)

**Correct interpretation:**

• Participants in the “Sex2_0” category have **0.489 times the odds** of the outcome compared to the reference category
• This means **51% lower odds** (1-0.489 = 0.511)
• **Statistically significant** (p = 0.012)

**Clinical translation:** “This sex category is approximately **half as likely** to experience the outcome compared to the reference group.”

2. Age 45: OR = 1.911 (p = 0.020)

**Correct interpretation:**

• Participants in the “Age 45” category have **1.911 times the odds** of the outcome compared to the reference category
• This means **91% higher odds** (1.911-1 = 0.911)
• **Statistically significant** association (p = 0.020)

**Clinical translation:** “This age group is approximately **twice as likely** (1.9 times) to experience the outcome compared to the reference age group.”

Coefficients values of the model and their statistical significance P-value were obtained by ‘Enter logistical regression method’. Logistic regression test was applied to evaluate the overall model fit and to test the significant coefficients. The B coefficient values were found to be (-0.716, 0.48), while the Wald Statistics values are (6.307, 5.438) for sex and age respectively. The exponentiated logistic coefficient (Exp (B)) shows the values of 0.489 for sex and 1.911 for age cut value 45 years old.

Our model in this work uses two indicators to measure model fineness percentage; Cox & Snell R Square (R ^2^ = 0.028) and Nagelkerke R Square (R ^2^ = 0.039). Although the R ^2^ values are too small, it indicated a weak relationship. This value explanted that out model contributes about 4% of the COVID-19 infection probability as illustrated in Table 4 above. It is important to mention that the Cox & Snell R Square indicator commonly produced underestimates the real value. ^36^^,^^37^

**Table 4.** Logistic regression model summary.

Step	-2 Log likelihood	Cox & Snell R Square	Nagelkerke R Square
1	480.878 ^a^	0.028	0.039

This research aimed to predict the probability of infection with COVID-19 in Karak city. Using the final logistic regression model data presented in Table 4 results, the formula of COVID-19 infection probability (P _infected_) is given as:

$$P_{\text{infected}}=\frac{e^{0.674-0.716*\text{Gender}+0.648*\mathrm{Age}}}{1+e^{0.674-0.716*\text{Gender}+0.648*\mathrm{Age}}}$$ (1)

The probability of infection can be calculated by substituting the values for sex and age in equation (1).

## Discussion

Our findings align with large-scale studies showing age as a consistent COVID-19 risk factor (Huyut et al., 2022), though our model’s predictive power is limited compared to comprehensive biomarker models achieving >95% accuracy. ^3^

According to the results obtained from Table 2 the number of infected women with COVID-19 was 223 (86.8%) and the number of infected men was 34 (13.2%). This difference is in agreement with the results of a study in the United States recorded from January to May 2020, where the number of infected women with COVID-19 was 51.1%, while the number of infected males was 48.9%. ^35^

In another study, infection rates for COVID-19 were reconstructed by age and sex using data from different European countries. ^38^ The results show in all the analyzed countries, the chance of infection with COVID-19 among women increases more sharply after age 20 until late 50s. ^38^

According to the results obtained from Table 2, the number of infected women with COVID-19 was 223 (86.8%) and the number of infected men was 34 (13.2%). This **significant female predominance differs markedly** from findings in the United States, where analysis of 1,320,488 confirmed COVID-19 cases from January 22, 2020 to May 30, 2020 found that the incidence of cases was similar between females and males Sex-differences in COVID-19 diagnosis, risk factors and disease comorbidities: A large US-based cohort study - PMC, and from contact-tracing studies showing 52.1% female and 47.9% male cases Sex-differences in COVID-19 diagnosis, risk factors and disease comorbidities: A large US-based cohort study - PMC. ^39^

However, our findings **align more closely** with European patterns documented by Sobotka *et al*, ^38^ who found that women substantially outnumber infected men among people of working age. The female face of COVID-19 infections in Europe | nexus in ten European countries, with women in their 20s showing the biggest gender gap in infections: on average only 64 men were infected per 100 infected women aged 20-29. The female face of COVID-19 infections in Europe | nexus. This **age-specific pattern** supports our findings, as the chance of infection with COVID-19 among women increases more sharply after age 20 until late 50s. The female face of COVID-19 infections in Europe | nexus across European populations.

These results support the ones obtained in our study since among the 188 (73.2%) participants aged <= 45 years infected with COVID-19, 167 (88.8%) of them were women and 21 (11.2%) were males.

Moreover, in this work among the former and current smokers infected with COVID-19, 222 (86.7%) (199 former and 23 current) are women, while 34 (13.3%) (12 former and 22 current) are men. These results are consistent with previous studies which recognized that one of the risk factors associated with COVID-19 is smoking. ^40^^–^^42^

Furthermore, in our sample 197 participants are suffering from chronic disease and have been infected with COVID-19. Previous study found that the increased COVID-19 severity and increased admittance to intensive care unit (ICU) were strongly correlated to preexisting chronic diseases. ^43^ Another study found that people with obesity, cardiovascular disease, hypertension, and neuromuscular illness were much more likely to experience severe pandemic influenza. ^44^ Moreover, Laires et al revealed that renal diseases, cardiovascular, and respiratory were associated with ICU admission and mortality within COVID-19 infected patients. ^45^

Among the 257 COVID-19 infected participants, 231 of them didn’t receive the yearly flu vaccine. Recent existing data in literatures indicates that influenza vaccination may lessen the COVID-19 clinical consequences. A meta-analysis study revealed a significant advantage for mechanical ventilation in COVID-19 individuals who had received an influenza vaccination over those who had not. ^46^ Another meta-analysis of observational studies included 290,327 participants claimed that receiving an influenza vaccination decreases the chance of COVID-19 infection which is in agreement with the findings of our study. ^47^ On the other hand, the relationship between the influenza vaccine and a decreased risk of COVID-19 negative outcomes was discussed by Fink *et al.* ^48^ This included the ability of live vaccines to activate trained innate immunity and produce known “off-target” protection against infections other than those specifically targeted by the vaccine. ^48^

The results of the exponentiated logistic coefficient show that the probability of women to be infected with COVID-19 is more than men for the same age. This result is in agreement with the results obtained from our model ( equation (1)) for calculating the probability of infection with COVID-19.

In equation (1) the sex variable labeled by (0) for women, (1) for men, while the age variable labeled by (0) if age less than or equal 45 years old and labeled (1) if age is more than 45 years old. Using equation (1) we can calculate the probability of a man aged 33 years old to be infected with COVID-19 by substituting numbers for age and sex. The results lead to P _infected_ = 0.4895. Since P _infected_ is less than 0.5, indicates the man is not infected. Repeating the same calculations for a woman with the same age result in P _infected_ = 0.6624, indicates the woman is infected with COVID-19.

### Limitations of the study

A notable limitation of this study was its brief lifespan. There was no more observation to validate the model. Furthermore, we are unable to extrapolate our results to the other cities in Jordan because we only evaluated people in Karak city. The observed R ^2^ values of 0.028-0.039 represent a significant limitation. In clinical medicine, R ^2^ values of 0.15-0.20 are considered suitable for clinical research, with our lower values reflecting the complex, multifactorial nature of COVID-19 pathogenesis. This limitation stems from our restricted variable set focusing primarily on demographic characteristics. We acknowledge that standardized disease severity classification using WHO or AIIMS guidelines was not implemented in the original study design.

## Conclusion

Given a person's age and sex, equation (1) presented in this study can be used to calculate the probability of infection with COVID-19 in Karak city. This statistical model can be used to forecast outbreak trends. This forecast could aid health-care management and policymakers in properly planning and allocating health-care resources.

## Data Availability

Fighsare: supplementary data.xlsx. https://doi.org/10.6084/m9.figshare.21829650.v1. ^49^ Figshare: questionnaire supplementary information.docx https://doi.org/10.6084/m9.figshare.21931731.v3. ^50^ Data are available under the terms of the Creative Commons Attribution 4.0 International license (CC-BY 4.0).
