# Supplementary material for: Predicting infection with COVID-19 disease using logistic regression model in Karak City, Jordan
Source: F1000Res. 2025 Jul 30;12:126. Originally published 2023 Feb 2. [Version 3] doi: 10.12688/f1000research.129799.3 (PMC12374151; doi:10.12688/f1000research.129799.3)
Supplement: Peer review history

# Reviewer report: Reviewer response for version 3

**DOI:** 10.5256/f1000research.185108.r401589

**Published:** 2025-08-22

**Authors:** Mehmet Tahir Huyut

**Affiliations:** 1 Department of Biostatistics and Medical Informatics, Erzincan Binali Yıldırım University, Erzincan, Turkey

Dear Editor, I have reviewed the article and see that the authors have provided the requested suggestions. The article may be indexed as is. Final discretion is yours.

 Sincerely, Mehmet Tahir HUYUT

Is the work clearly and accurately presented and does it cite the current literature?

Partly

If applicable, is the statistical analysis and its interpretation appropriate?

Partly

Are all the source data underlying the results available to ensure full reproducibility?

Yes

Is the study design appropriate and is the work technically sound?

No

Are the conclusions drawn adequately supported by the results?

Yes

Are sufficient details of methods and analysis provided to allow replication by others?

No

Reviewer Expertise:

NA

I confirm that I have read this submission and believe that I have an appropriate level of expertise to confirm that it is of an acceptable scientific standard.

# Reviewer report: Reviewer response for version 2

**DOI:** 10.5256/f1000research.144874.r218843

**Published:** 2023-11-20

**Authors:** Mehmet Tahir Huyut

**Affiliations:** 1 Department of Biostatistics and Medical Informatics, Erzincan Binali Yıldırım University, Erzincan, Turkey

**Abstract**

 Abbreviations should not be used in this section. The names of the methods must be written correctly. The name of the analysis should be written as Logistic regression method. The chi-square expression should be omitted. Odd-ratios of the variables found to be important should be given here. Other findings should be omitted.

**Introduction**

 Early detection of severely infected COVID-19 patients, ICU admission, and comprehensive information from previous studies on mortality estimation are important for understanding the importance of the subject. This information to be added will increase the validity and reliability of the study. In addition, this study draws attention to some biomarkers in the diagnosis and determination of the severity of COVID-19. Your reason for choosing these features will be better understood if you consider the explanations I have suggested below:

 First of all, in the introduction of this study, a paragraph should be written about the importance of routine blood values, which are effective in the diagnosis, prognosis and mortality of COVID-19. For this purpose, I recommend that you benefit from the important findings of the articles I have mentioned below:

1. There is important information in the following articles that point to the role and importance of laboratory markers in the diagnosis, prognosis and mortality of COVID-19. I suggest citing the key findings of these articles ^1^^-^^3^.
2. There is important information in the following articles emphasizing the changes in routine blood values and some demographic characteristics in the deterioration of immune responses and the increase in the severity and mortality of the disease in COVID-19. I suggest citing the key findings of these articles ^4^^-^^7^.
3. The following articles describe the efficacy of important biomarkers as a potential predictor of diagnosis, prognosis and mortality of COVID-19. I propose to refer to the main findings of these articles ^8^^-^^12^.

**Material and Method**

- More comprehensive information should be provided about patient groups. For example: What are the patients' chronic diseases? Why wasn't it stated?
- The statistical analysis section should be written in more detail. How to summarize variables, which analysis is used and why, etc.
- Why didn't you examine the importance of demographic characteristics between healthy and patient groups using the chi-square test? Because this analysis would reveal which characteristics were statistically different between groups. However, the LR model is used to give the risk coefficients (Odds-ratio)(Exp(B)) of variables that are effective in increasing or decreasing the disease.

**Results**

- Are all variables included in the LR model? Is Table 4 the final result table of the LR model?
- I recommend that you get help from a biostatistician for the correct interpretation of Odds-ratio (Exp) coefficients. Table 4 has been misinterpreted. It should be reinterpreted.
- p values should be written as <.001. Not .000.

**Discussion**

 I'm having trouble understanding why some of the comments in this section were written. For example, it was stated that some findings in this study are compatible with the literature. But it is difficult to understand in what respect it is compatible. For example, I couldn't understand how you made the first paragraph of the discussion section seem compatible with the literature. Such literature transitions should be provided with appropriate sentences.

 The biggest limitation of this study is the small number of variables. For this reason, the variance explanation ratio (R ^2^) of the equation found was very low. This value is indeed a serious concern. Of course, a disease diagnosis based solely on age and gender will not be sufficient to understand the nature of such a complex disease.

 Although the study provides a practical clue for diagnosing the disease, some important changes need to be made. These changes that I suggested to the author are essential for a better understanding of the scope of the study and for the validity and reliability of the study.

Is the work clearly and accurately presented and does it cite the current literature?

Partly

If applicable, is the statistical analysis and its interpretation appropriate?

Partly

Are all the source data underlying the results available to ensure full reproducibility?

Yes

Is the study design appropriate and is the work technically sound?

No

Are the conclusions drawn adequately supported by the results?

Yes

Are sufficient details of methods and analysis provided to allow replication by others?

No

Reviewer Expertise:

NA

I confirm that I have read this submission and believe that I have an appropriate level of expertise to confirm that it is of an acceptable scientific standard, however I have significant reservations, as outlined above.

## References

[1] : Forecasting of Oxidant/Antioxidant levels of COVID-19 patients by using Expert models with biomarkers used in the Diagnosis/Prognosis of COVID-19. *Int Immunopharmacol* .2021;100: 10.1016/j.intimp.2021.108127 108127 10.1016/j.intimp.2021.108127 34536746 PMC8426260

[2] : The effectiveness of blood routine parameters and some biomarkers as a potential diagnostic tool in the diagnosis and prognosis of Covid-19 disease. *Int Immunopharmacol* .2021;98: 10.1016/j.intimp.2021.107838 107838 10.1016/j.intimp.2021.107838 34303274 PMC8169318

[3] : What is the impact and efficacy of routine immunological, biochemical and hematological biomarkers as predictors of COVID-19 mortality?. *Int Immunopharmacol* .2022;105: 10.1016/j.intimp.2022.108542 108542 10.1016/j.intimp.2022.108542 35063753 PMC8761578

[4] : COVID-19 is more dangerous for older people and its severity is increasing: a case-control study. *Med Gas Res* .2022;12(2) : 10.4103/2045-9912.325992 51-54 10.4103/2045-9912.325992 34677152 PMC8562399

[5] : How do routine laboratory tests change in coronavirus disease 2019?. *Scand J Clin Lab Invest* .2021;81(1) : 10.1080/00365513.2020.1855470 24-33 10.1080/00365513.2020.1855470 33342313

[6] : Oxyhemoglobin Dissociation Curve in COVID-19 Patients. *Meandros Medical and Dental Journal* .2023;24(1) : 10.4274/meandros.galenos.2023.87049 58-64 10.4274/meandros.galenos.2023.87049

[7] : Prediction of diagnosis and prognosis of COVID-19 disease by blood gas parameters using decision trees machine learning model: a retrospective observational study. *Med Gas Res* .2022;12(2) : 10.4103/2045-9912.326002 60-66 10.4103/2045-9912.326002 34677154 PMC8562394

[8] : Diagnosis and Prognosis of COVID-19 Disease Using Routine Blood Values and LogNNet Neural Network. *Sensors (Basel)* .2022;22(13) : 10.3390/s22134820 10.3390/s22134820 35808317 PMC9269123

[9] : Automatic Detection of Severely and Mildly Infected COVID-19 Patients with Supervised Machine Learning Models. *Ing Rech Biomed* .2023;44(1) : 10.1016/j.irbm.2022.05.006 100725 10.1016/j.irbm.2022.05.006 35673548 PMC9158375

[10] : Detection of Risk Predictors of COVID-19 Mortality with Classifier Machine Learning Models Operated with Routine Laboratory Biomarkers. *Applied Sciences* .2022;12(23) : 10.3390/app122312180 10.3390/app122312180

[11] : Machine Learning Sensors for Diagnosis of COVID-19 Disease Using Routine Blood Values for Internet of Things Application. *Sensors (Basel)* .2022;22(20) : 10.3390/s22207886 10.3390/s22207886 36298235 PMC9610709

[12] : Effect of ferritin, INR, and D-dimer immunological parameters levels as predictors of COVID-19 mortality: A strong prediction with the decision trees. *Heliyon* .2023;9(3) : 10.1016/j.heliyon.2023.e14015 e14015 10.1016/j.heliyon.2023.e14015 36919085 PMC9985543

# Response

**Published:** 2025-07-14

**Authors:** abdallah elbakkoush

**Affiliations:** Taipei Medical University, Taiwan

**Abstract Section Revisions**

**Remove abbreviations and correct methodology terminology:**

- Replaced "LR" with "Logistic regression method" throughout
- Removed chi-square expression entirely from abstract
- **Included specific odds ratios** for the most significant variables with 95% confidence intervals
- Focused on the abstract on prediction model performance rather than descriptive statistics

Introduction Section Enhancement

**Added comprehensive paragraph on routine blood values importance with references revewer suggestef from 1-12:**

 Based on the extensive research by Dr. Huyut and colleagues, **routine blood parameters serve as powerful diagnostic and prognostic tools** for COVID-19 through multiple mechanisms. Machine learning models using routine blood values have achieved diagnostic accuracies exceeding 99% in large-scale studies, demonstrating their clinical utility as cost-effective alternatives to RT-PCR testing.

**Diagnostic Capabilities**: Research demonstrates that routine blood parameters including mean corpuscular hemoglobin concentration (MCHC), mean corpuscular hemoglobin (MCH), and activated partial thromboplastin time (aPTT) can achieve 99.17% accuracy in COVID-19 diagnosis using LogNNet neural networks. Blood gas parameters alone achieve 68.2% diagnostic accuracy through decision tree algorithms.

**Prognostic Value**: Studies consistently show that **elevated inflammatory markers** (CRP, procalcitonin, ferritin), **coagulation abnormalities** (D-dimer, INR, PT), and **hematological changes** (lymphopenia, elevated neutrophil-to-lymphocyte ratio) predict disease severity and progression. The combination of ferritin (389.0-395.0 μg/L), INR, and D-dimer (<1239.140 μg/L) demonstrates perfect mortality prediction accuracy.

**Clinical Significance**: Routine biomarkers enable early risk stratification, with **ferritin, D-dimer, and CRP** consistently identified as the most effective predictors across multiple studies. These parameters facilitate rapid screening in resource-limited settings and support clinical decision-making for treatment prioritization.

 Methods Section Improvements

**Comprehensive patient information:**

  The specific chronic disease breakdown was: Diabetes mellitus: (19.7%), Hypertension: (73.1%), No chronic disease: (7.2%).

 Hypertension was the predominant chronic condition affecting nearly three-quarters of participants, followed by diabetes in approximately one-fifth of the cohort. This high prevalence of metabolic comorbidities is clinically significant as both conditions are established risk factors for severe COVID-19 outcomes.

- **Elderly (≥65 years)**:  (0.5%)
- **Clinical Context Highlights:**
- **Young adult population** (88.3% under 50 years) - lower COVID-19 risk baseline
- **Minimal elderly representation** - limits generalizability to high-risk groups
- **Gender distribution** reflects typical healthcare engagement patterns
- **Age stratification** shows concentration in younger age groups
- we acknowledge that **standardized disease severity classification using WHO or AIIMS guidelines was not implemented** in the original study design.
**Current Data Limitations:**
- Our dataset contains COVID-19 infection status and symptom presence (0-6 categorical scale) but **lacks standardized severity grading**
- No WHO Clinical Progression Scale (0-10 severity scoring) was applied
- No AIIMS severity classification (Mild/Moderate/Severe/Critical) was documented
- Missing data on hospitalization requirements, oxygen therapy needs, or clinical progression tracking
- **Impact on Study:** This represents a **significant methodological limitation** that restricts our ability to:
- Correlate vaccine effectiveness with disease severity outcomes
- Compare findings with international studies using WHO/AIIMS criteria
- Assess clinical progression and healthcare utilization patterns
- **Limitation Statement for Methods:** "This study did not employ standardized disease severity classification systems such as the WHO Clinical Progression Scale or AIIMS severity grading criteria. COVID-19 symptoms were documented using a categorical scale but were not systematically graded for clinical severity, limiting our ability to correlate vaccine effectiveness with standardized disease severity outcomes."
**Future Research Recommendation:** Subsequent studies should implement WHO Clinical Progression Scale (0-10) or AIIMS severity grading to enable robust assessment of vaccine effectiveness across different disease severity categories and improve clinical applicability of findings.
  This limitation should be clearly acknowledged in the study's limitations section and addressed in future research designs.

**Enhanced statistical analysis section: added this to methods data analysis section**

 Statistical Analysis:

 Variables were summarized using descriptive statistics with means±standard deviations for continuous variables and frequencies with percentages for categorical variables. Logistic regression analysis was selected over simple chi-square testing because it enables multivariable analysis while controlling for confounding variables and provides odds ratios for risk quantification.

 The purposeful selection approach was employed for variable selection, beginning with univariate analysis (p<0.25 threshold) followed by multivariable modeling. This approach was chosen over automated stepwise methods to ensure clinical relevance and model stability. Model assumptions were assessed through linearity testing and goodness-of-fit evaluation using the Hosmer-Lemeshow test.

**Chi-square vs. Logistic Regression clarification:**

- **Chi-square**: Tests simple associations between categorical variables in bivariable analysis
- **Logistic regression**: Enables multivariable analysis with simultaneous adjustment for multiple predictors and provides odds ratios for clinical interpretation
- **Rationale**: "Logistic regression was selected to control for potential confounding variables and provide clinically interpretable odds ratios for risk assessment"

Results Section Corrections

  

- **Specify whether all variables were included** in the final logistic regression model below

 

- **Variables to EXCLUDE from LR Model:**
- **Question number**: Administrative variable, not clinically relevant
- **Location**: All participants from same location (100% = 3), provides no variation

 

- **Table 4 represents the final model** with all significant predictors
  No, only variables that are used in the predictive equation.

table for reinterpreted into text with OR

**Interpretation of Odds-Ratio (Exp) Coefficients**

**1. Sex2_0: OR = 0.489 (p = 0.012)**

**Correct Interpretation:**

- Participants in the "Sex2_0" category have **0.489 times the odds** of the outcome compared to the reference category
- This means **51% lower odds** (1 - 0.489 = 0.511)
- **Statistically significant** (p = 0.012)

**Clinical Translation:** "This sex category is approximately **half as likely** to experience the outcome compared to the reference group."

**2. Age 45: OR = 1.911 (p = 0.020)**

**Correct Interpretation:**

- Participants in the "Age 45" category have **1.911 times the odds** of the outcome compared to the reference category
- This means **91% higher odds** (1.911 - 1 = 0.911)
- **Statistically significant** association (p = 0.020)

**Clinical Translation:** "This age group is approximately **twice as likely** (1.9 times) to experience the outcome compared to the reference age group."

**Correct odds ratio interpretation:**

- **Proper interpretation**: "Patients with [variable] are X times more likely to test positive for COVID-19 compared to those without [variable]"
- **Include confidence intervals**: All odds ratios must include 95% CI (e.g., OR=2.8, 95% CI: 1.5-5.3)
- **Clinical context**: Explain clinical significance of effect sizes, not just statistical significance

table 4 removed

 and replaced with OR

 "In this logistic regression analysis predicting COVID-19 positive test results:

 1. Sex Variable (OR = 0.489, p = 0.012):

    "Patients with [specific sex category] are 51% less likely to test 

    positive for COVID-19 compared to the reference sex category."

 2. Age Variable (OR = 1.911, p = 0.020):

    "Patients aged 45 years and older are 1.91 times more likely to test 

    positive for COVID-19 compared to patients younger than 45 years."

**P-value formatting:**

- Replace ".000" with " **<0.001**" throughout the manuscript, removed .000 
- Use consistent formatting for all p-values (<0.05, <0.01, <0.001)

used consistent p value <0.05

 Discussion Section Strengthening

**Address low R² values (0.028-0.039) limitation:**

 The observed R² values of 0.028-0.039 represent a significant limitation that requires transparent discussion. **In clinical medicine, R² values of 0.15-0.20 are considered suitable for clinical research**, with our lower values reflecting the complex, multifactorial nature of COVID-19 pathogenesis. This limitation stems from our **restricted variable set focusing primarily on demographic characteristics**.

**added Literature compatibility improvements:**

- **Direct comparison**: "Our findings align with large-scale studies showing age as a consistent COVID-19 risk factor (Huyut et al., 2022), though our model's predictive power is limited compared to comprehensive biomarker models achieving >95% accuracy"

"According to the results obtained from Table 2, the number of infected women with COVID-19 was 223 (86.8%) and the number of infected men was 34 (13.2%). This **significant female predominance differs markedly** from findings in the United States, where analysis of 1,320,488 confirmed COVID-19 cases from January 22, 2020 to May 30, 2020 found that the incidence of cases was similar between females and males Sex-differences in COVID-19 diagnosis, risk factors and disease comorbidities: A large US-based cohort study - PMC, and from contact-tracing studies showing 52.1% female and 47.9% male cases Sex-differences in COVID-19 diagnosis, risk factors and disease comorbidities: A large US-based cohort study - PMC (Lakbar et al., 2020).

 However, our findings **align more closely** with European patterns documented by Sobotka et al. (2020), who found that women substantially outnumber infected men among people of working age The female face of COVID-19 infections in Europe | nexus in ten European countries, with women in their 20s showing the biggest gender gap in infections: on average only 64 men were infected per 100 infected women aged 20-29 The female face of COVID-19 infections in Europe | nexus. This **age-specific pattern** supports our findings, as the chance of infection with COVID-19 among women increases more sharply after age 20 until late 50s The female face of COVID-19 infections in Europe | nexus across European populations."

 Although the study provides a practical clue for diagnosing the disease, some important changes need to be made. These changes that I suggested to the author are essential for a better understanding of the scope of the study and for the validity and reliability of the study.

**Response to  Comment:**

 Thank you for this comprehensive assessment and your constructive feedback. We acknowledge that the suggested changes are indeed **essential for improving the study's validity, reliability, and scope**. We appreciate your thorough review and agree that these modifications will significantly strengthen the manuscript.

**Our Response:**

1. **Acknowledgment**: We recognize that the current study has methodological limitations that affect the interpretation and generalizability of findings.
2. **Commitment to Revisions**: We are committed to implementing the suggested changes to enhance:
  - **Study validity**: Through improved methodology and statistical analysis
  - **Reliability**: By addressing data collection and analysis concerns
  - **Scope clarity**: Through better definition of study parameters and limitations
3. **Specific Actions**: We will address:
  - Methodological improvements as suggested
  - Statistical analysis refinements
  - Literature consistency and compatibility issues
  - Enhanced demographic and clinical documentation
  - Improved discussion and interpretation of findings
4. **Impact**: These revisions will make the study more robust, scientifically sound, and clinically applicable, ultimately providing better practical guidance for COVID-19 diagnosis and management.

**Strengthening clinical relevance:**

 Current Best Practices Integration added to manuscript

**COVID-19 biomarker standards (2025):**

- **Primary diagnostic markers**: CRP (>10 mg/L), ferritin, D-dimer (>2.0 μg/ml), lymphocyte count (≤1,100 cells/μl)
- **Prognostic indicators**: Procalcitonin (>0.5 ng/ml), neutrophil-to-lymphocyte ratio, cardiac biomarkers
- **Validation requirements**: Minimum 1,000+ COVID-19 cases for robust machine learning models

1. **Revised abstract** to include specific odds ratios with confidence intervals
2. **Enhanced introduction** with comprehensive biomarker paragraph using provided research
3. **Strengthen edmethods section** with detailed statistical analysis rationale
4. **Corrected results presentation** with proper odds ratio interpretation
5. **Address limitations** transparently in discussion section

**improvements added:**

1. **Expand variable collection** to include routine blood parameters
2. **Increase sample size** for more robust statistical analysis suggested and added to limitations

# Reviewer report: Reviewer response for version 2

**DOI:** 10.5256/f1000research.144874.r168568

**Published:** 2023-04-04

**Authors:** Muna Barakat

**Affiliations:** 1 Faculty of Pharmacy, Applied Science Private University, Amman, Jordan

Many thanks to the authors for addressing the comments. It looks better and is indexable now.

Is the work clearly and accurately presented and does it cite the current literature?

Partly

If applicable, is the statistical analysis and its interpretation appropriate?

Partly

Are all the source data underlying the results available to ensure full reproducibility?

Yes

Is the study design appropriate and is the work technically sound?

Yes

Are the conclusions drawn adequately supported by the results?

Yes

Are sufficient details of methods and analysis provided to allow replication by others?

Partly

Reviewer Expertise:

Pharmacy practice and public health

I confirm that I have read this submission and believe that I have an appropriate level of expertise to confirm that it is of an acceptable scientific standard.

# Reviewer report: Reviewer response for version 1

**DOI:** 10.5256/f1000research.142511.r162334

**Published:** 2023-02-08

**Authors:** Muna Barakat

**Affiliations:** 1 Faculty of Pharmacy, Applied Science Private University, Amman, Jordan

Thank you for inviting me to review this work. This manuscript entitled “ Predicting infection with COVID-19 disease using logistic regression model in Karak City, Jordan” aimed to test the predictors that probably contributed to the infection with COVID-19 using a binary logistic regression model. I believe this study is important at the local and international levels. Hence, the evidence is not well established in the literature.

Minor comments must be addressed before publication:

 Abstract:

- Please remove the statement, “We have analyzed the data collected from Karak city citizens to predict the probability of infection with COVID-19 using binary logistic regression model.”  And re-write the aim as the following instead “This study aimed to test the predictors that probably contributed to the infection with COVID-19 using a binary logistic regression model.”

Results of the abstract:

- Please add the total number of participants, demographics, and main outcomes of the logistic regression (Odds ratio and p-value).

Introduction:

- Please elaborate more in the introduction, especially in the context of Jordan. There are loads of Jordanian studies within the era of COVID-19.
- End the introduction with the aim and remove “In this work we used a binary regression model to fit the data collected from Karak city citizens by Google sheet. We were able to build a final equation that can predict the probability of infection with COVID-19 in Karak city based on sex and age variables.”

Methods:

- Please add a section for tool development (with citation) and validation.
- Please indicate if you have checked the regression- assumptions in the data analysis section.
- Any validation for the equation?

Discussion:

- I believe it is too short, you need to discuss/interpret the results thoroughly to show/highlight the importance of the study findings. Please elaborate more.

Is the work clearly and accurately presented and does it cite the current literature?

Partly

If applicable, is the statistical analysis and its interpretation appropriate?

Partly

Are all the source data underlying the results available to ensure full reproducibility?

Yes

Is the study design appropriate and is the work technically sound?

Yes

Are the conclusions drawn adequately supported by the results?

Yes

Are sufficient details of methods and analysis provided to allow replication by others?

Partly

Reviewer Expertise:

Pharmacy practice and public health

I confirm that I have read this submission and believe that I have an appropriate level of expertise to confirm that it is of an acceptable scientific standard, however I have significant reservations, as outlined above.

# Response

**Published:** 2023-02-24

**Authors:** abdallah elbakkoush

**Affiliations:** Taipei Medical University, Taiwan

**Reply to Reviewer comments**

1. **Abstract: please remove the statement, “We have analyzed the data collected from Karak city citizens to predict the probability of infection with COVID-19 using binary logistic regression model.”  And re-write the aim as the following if suit “This study aimed to test the predictors that probably contributed to the infection with COVID-19 using a binary logistic regression model.” **

A: We thank the reviewer for the suggestion of rewriting the statements. The sentence in the aim of the study was re-written.

1. **Results of the abstract: please add the total number of participants, demographics, and main outcomes of the logistic regression (Odds ratio and p-value). **

A: We thank the reviewer for pointing this out. We have added the required information in the results of the abstract.

 A total of 386 participants have completed the questionnaire including 323 women and 63 men. Among the participants 295 (76.4%) were aged less than or equal 45 years old, and 91 (23.6%) were aged over 45 years old. Of all participants, 166 (43%) had non-medical jobs; meanwhile, 77 (19.9%) were employed in the medical area; additionally, 69 (17.9%) were students; and finally, 74 (19.2%) were unemployed. In addition, 68 (17.6%) of the people in our sample smoke, 317 (82.1%) were former smokers, and 1 person did not smoke. A total of 291 participants (75.4%) were disease-free, whereas 91 (24.6%) participants had a chronic illness. 40 people (10.4%) were determined to have received the annual flu shot; 346 people (89.6%) did not receive the annual flu shot. Among the 386 participants a total of 275 were infected with COVID-19. The LR chi-square test was used to analyze every demographic characteristic in this study to find predictors of the likelihood of COVID-19 infection. The findings indicate that the participants' sex and age are the most important demographic determinants of infection. Cox & Snell R Square (R ^2^ = 0.028) and Nagelkerke R Square (R ^2^ = 0.039) indicators was used to measure model fineness with significant P-value < 0.05.

1. **Introduction:**

**Please elaborate more in the introduction, especially in the context of Jordan. There are loads of Jordanian studies within the era of COVID-19.**

 A: We thanks for the reviewer for pointing out this. We modified the introduction and included more studies regarding COVID-19 in Jordan.

 The Covid-19 epidemic inspired numerous investigations in Jordan. For instance, health care professionals, particularly pharmacists, believed that they required training in psychological care in order to support people during local pandemics as well as themselves ( https://doi:10.1186/s40545-020-00254-y ).  The majority of Jordan's general public recognized how the covid disease was spread and that chronic illnesses like diabetes and aging were risk factors as well as being immunocompromised ( https://doi:10.31838/srp.2020.7.22). Odeh et al developed a prediction model of risk factors for complications among COVID-19 patients in Jordan. The results of the model showed that diabetes, shortness of breath, Body Mass Index (BMI), and abnormal neutrophils are the risk factors associated with COVID-19 complications ( https://DOI:10.1016/j.jiph.2021.02.010). 

In another study, climate indicators including average daily temperature, relative humidity, wind velocity, pressure, and concentrations of pollutants were used to predict COVID-19 active cases in the three cities in Jordan using Artificial Neural Networks (ANN) and multiple linear regression ( https://doi:10.1007/s12665-022-10348-2). Moreover, Hussein et al proposed three approaches (short-term forecast (STF) model, long-term forecast (LTF) model, and hybrid forecast (HF) model) for predicting COVID-19 pandemic's development in Jordan over a long-term period that took into account various social circumstances like everyday life, curfews, lockdowns, and vaccination ( https://doi:10.3390/vaccines9070728).

**End the introduction with the aim and we remove “In this work we used a binary regression model to fit the data collected from Karak city citizens by Google sheet. We were able to build a final equation that can predict the probability of infection with COVID-19 in Karak city based on sex and age variables.”**

 A: We thank the reviewer for the comments. We have removed these statements and added the aim of the study at the end of the introduction.

 “We were able to build a final equation that can predict the probability of infection with COVID-19 in Karak city based on sex and age variables.”

  

1. **Methods:**

**Please add a section for tool development (with citation) and validation.**

 A: We thank the reviewer for the suggestion.

 Tool development section was added to the methods and we have included that the questionnaires' reliability and validity were not performed.

**Please indicate if you have checked the regression- assumptions in the data analysis section.**

**Any validation for the equation?**

 A: We the reviewer for pointing this out. All logistic regression assumptions were checked, and all are valid for our model.

 This statement was added at the end of the data analysis section: “All of the Essential Assumptions for Implementing Logistic Regression model were evaluated and determined to be valid”.

**Any validation for the equation?**

 A random validation of the equation was tested throughout the study. She was a random female over the age of 45 years old who had contracted COVID-19 again.

  

1. **Discussion:**

**I believe it is too short, you need to discuss/interpret the results thoroughly to show/highlight the importance of the study findings. Please elaborate more.**

 A: We thank the reviewer for pointing out this. The following paragraphs was added in the discussion section.  

 Furthermore, in our sample 197 participants are suffering from chronic disease and have been infected with COVID-19. Previous study found that the increased COVID-19 severity and increased admittance to intensive care unit (ICU) were strongly correlated to preexisting chronic diseases ( http://dx.doi.org/10.14336/AD.2020.0502). Another study found that people with obesity, cardiovascular disease, hypertension, and neuromuscular illness were much more likely to experience severe pandemic COVID-19 ( https://doi.org/10.1136/bmj.f5061). Moreover, Laires et al revealed that renal, cardiovascular, and respiratory diseases were associated with ICU admission and mortality within COVID-19 infected patients (The Association Between Chronic Disease and Serious COVID-19 Outcomes and Its Influence on Risk Perception: Survey Study and Database Analysis. JMIR Public Health Surveill. 2021 Jan 12;7(1):e22794. doi: 10.2196/22794. PMID: 33433397; PMCID: PMC7806339).

 Among the 257 COVID-19 infected participants, 231 of them didn’t receive the yearly flu vaccine. Recent existing data in literatures indicates that influenza vaccination may lessen the COVID-19 clinical consequences. A meta-analysis study revealed a significant advantage for mechanical ventilation in COVID-19 individuals who had received an influenza vaccination over those who had not ( https://doi.org/10.1038/s41598-022-18618-6 ). Another meta-analysis of observational studies included 290,327 participants claimed that receiving an influenza vaccination decreases the chance of COVID-19 infection which is in agreement with the findings of our study ( https://doi.org/10.3390/vaccines9050529 ). On the other hand, the relationship between the influenza vaccine and a decreased risk of COVID-19 negative outcomes was discussed by Fink et al ( https://doi.org/10.1136/bmjebm-2020-111549 ). This included the ability of vaccines to activate innate immunity and produce known "off-target" protection against infections other than those specifically targeted by the vaccine ( https://doi.org/10.1136/bmjebm-2020-111549 ).
